# Viral Etiology and Clinical Profiles of Children with Severe Acute Respiratory Infections in China

**DOI:** 10.1371/journal.pone.0072606

**Published:** 2013-08-22

**Authors:** Chen Zhang, Na Zhu, Zhengde Xie, Roujian Lu, Bin He, Chunyan Liu, Xuejun Ma, Wenjie Tan

**Affiliations:** 1 Key Laboratory of Medical Virology, Ministry of Health, National Institute for Viral Disease Control and Prevention, China Centers for Disease Control and Prevention (CDC), Beijing, China; 2 Key Laboratory of Major Diseases in Child and National Key Discipline of Pediatrics, Beijing Pediatric Research Institute, Beijing Children’s Hospital, Capital Medical University, Beijing, China; 3 Huainan Centers for Disease Control and Prevention (CDC), Huainan, Anhui, China; University of Georgia, United States of America

## Abstract

**Background:**

No comprehensive analysis is available on the viral etiology and clinical characterization among children with severe acute respiratory infection (SARI) in China during 2009 H1N1 pandemic and post-pandemic period.

**Methods:**

Cohort of 370 hospitalized children (1 to 72 months) with SARI from May 2008 to March 2010 was enrolled in this study. Nasopharyngeal aspirate (NPA) specimens were tested by a commercial assay for 18 respiratory viral targets. The viral distribution and its association with clinical character were statistically analyzed.

**Results:**

Viral pathogen was detected in 350 (94.29%) of children with SARI. Overall, the most popular viruses were: enterovirus/rhinovirus (EV/RV) (54.05%), respiratory syncytial virus (RSV) (51.08%), human bocavirus (BoCA) (33.78%), human parainfluenzaviruse type 3 (PIV3) (15.41%), and adenovirus (ADV) (12.97%). Pandemic H1N1 was the dominant influenza virus (IFV) but was only detected in 20 (5.41%) of children. Moreover, detection rate of RSV and human metapneumovirus (hMPV) among suburb participants were significantly higher than that of urban area (P<0.05). Incidence of VSARI among suburb participants was also significant higher, especially among those of 24 to 59 months group (P<0.05).

**Conclusion:**

Piconaviruses (EV/RV) and paramyxoviruses are the most popular viral pathogens among children with SARI in this study. RSV and hMPV significantly increase the risk of SARI, especially in children younger than 24 months. Higher incidence of VSARI and more susceptibilities to RSV and hMPV infections were found in suburban patients.

## Introduction

Acute respiratory infection (ARI) is the leading causes of children death globally [1–5]. Even in developed country, severe acute respiratory infection (SARI), with its high mortality and morbidity among children younger than 5, impose great burden on the society [2,3].

Lacking of etiologic diagnosis is the main reason that more than 50% of ARI cases suffered unnecessary or inappropriate prescription of antibiotics, since most acute respiratory tract infections are caused by viruses [3]. This often leads to severe consequence such as high rate of resistance [6], especially in those with severe acute respiratory infection (SARI), which frequently happens in virus-infected children [3]. Therefore etiology studies, especially those of viruses, have been performed to help diagnosis and proper antiviral treatment of SARI [7,8], as well as prevention of nosocomial infections among in-patients [9].

Diagnosis of ARI is complicated by the wide range of potential pathogens that can present with similar clinical symptoms [10,11]. In recent years, the introduction of nucleic acid based diagnostic tests has markedly improved our ability in understanding viral etiology among ARI patients [12]. xTAG® RVP FAST, a test based on multiplex PCR and Luminex Molecular Diagnostics Universal Array that detects 18 most commonly seen viral targets in respiratory infection, is approved by the US Food and Drug Administration (FDA) and has shown improved viral detection ability as compared to traditional methods like direct fluorescent antibody (DFA) and culture methodology [13–19].

Shortly after the advent of severe acute respiratory syndrome (SARS) and the avian influenza, the emergence of the influenza virus A (H1N1) 2009 pandemic caused significant vibrations to the public health authorities and stressed the health systems worldwide. This highlighted our weaknesses regarding the diagnosis and assessment of SARI. China is the biggest developing country with largest population of child. However, we have found no published case-control studies regarding comprehensive viral etiology and clinical characterization of children SARI using commonly acknowledged reliable test in China. To better understand the role of respiratory viruses in children with SARI during 2009 H1N1 pandemic and postpandemic era, and help diagnosis and antiviral treatment, we conducted a comprehensive evaluation of viral etiology and clinical characterization among hospitalized children with SARI admitted to the Beijing Children Hospital from May 2008 to March 2010. This study has increased our knowledge on the management of SARI and community-acquired pneumonia.

## Methods

### Ethics Statement

The study protocol was approved by the Institutional Review Board of National Institute for Viral Disease Control and Prevention, China CDC, and the Scientific Committee of the Beijing Pediatric Research Institute, and the Ethical Review Committee of Beijing Children’s Hospital. Individual written informed consent was obtained from the parents or guardians of all participants.

### Participants and Clinical Definitions

All participants in this study were inpatients from Beijing Children’s Hospital between May 2008 and March 2010, diagnosed as SARI based on clinical grounds recommended by the World Health Organization (WHO) (20). Eligibility and classification of the clinical syndromes of SARI were determined from individual’s original record of medical history and examination. The criteria of hospitalized patient inclusion were: sudden onset of fever >38^o^C and cough or sore throat and difficulty breathing (dyspnea, oxygen saturation < 90%). Additional criteria were a normal or low leukocyte count, or lower chest wall indrawing.

Among these SARI cases, 120 were defined as very severe acute respiratory infection (VSARI) cases with any of the following criteria: 1) incidence of complication such as vomit or diarrhea, 2) respiratory failure or anhelation or heart failure, 3) ICU admissions.

Nasopharyngeal aspirate (NPA) or blood or induced sputum (IS) was collected from the patients at the first day of admission and transferred into virus transport medium and stored at -70 ^o^C until tested. Demographic information and medical test result were collected with standardized forms.

### Laboratory Methods

Nucleic acid was extracted from 200 µL of the virus transport medium using QIAamp MinElute Virus Spin Kit (Qiagen, Mississauga, Ontario, Canada) according to the manufacturer’s instructions. 10 µL of the nucleic acids were tested using xTAG® RVP FAST assay according to the manufacturer’s instructions (Abbott Molecular Inc., USA) and analyzed on Bio-Plex 200 system (Bio-Rad Laboratories, Inc., USA).

The RVP Fast assay simultaneously detects the following viruses: respiratory syncytial virus (RSV); influenza(IFV) A (H1, H3, and H5) and B viruses; parainfluenza viruse (PIV) 1, 2, 3, and 4; human metapneumovirus (hMPV); adenovirus(ADV); piconavirus(PIC) which includes enterovirus (EV) and rhinovirus (RV); human coronaviruse(HCoV) NL63, HKU1, 229E, and OC43; and human bocavirus(BoCA). The assay also includes an internal positive control added to each specimen at the extraction stage (*Escherichia coli* phage MS2 RNA) and a positive run control that is added to each run (bacteriophage lambda DNA).

### Statistical Analysis

Comparisons between urban and suburb patients, as well as severe and very severe infection, were performed using chi-square test. Correlation of virus detected and clinical signs or diagnosis was performed using binary logistic regression of SPSS statistics 17.0. Comparison of continuous variables like body temperature was conducted using variance analysis.

## Results

### Characteristics of Study Participants

370 hospitalized children with SARI from Beijing Children’s Hospital between May 2008 and March 2010 were selected in this study (Table 1). The mean age of study participants was 13.08(1 to 93) months. Most cases (82.7%) were under 24 months. 60.27% participants were male. 65.14% participants were from urban area, 34.86% from suburb or rural area of China (Anhui, Hebei, Henan, Liaoning, Shandong, Shanxi, Sichuan provinces and Inner Mongolia). Among these patients, 34.43% suffered VSARI. At least one respiratory virus was detected in 94.29%(95% confidence interval: 91.4-96.8%) of NPS from enrolled participants. Also no case was positive of *Mycobacterium tuberculosis* based on clinical finding and further IS culture (data not shown).

**Table 1:** Demographics and Viral Etiology of All Participants (N=370).

**Characteristics**		**No.**	**%(95% CI)**
**Gender**	Male	223	60.27
	Female	147	39.73
**Age mean(range)**		13.08 (1-93)	
**Area**	Urban	241	65.14
	Suburb or Rural	129	34.86
**Age(month)**			
	0-5	151	40.81
	6-23	155	41.89
	24-59	57	15.41
	> = 60	7	1.89
**Severity**	VSARI	120	32.43
**Viruses Detected**	**Any virus**	350(N=370)	94.29 (91.4-96.8)
**Orthomyxoviuses**		26	7.03
	IFVA-pH1N1	20	5.41
	IFVA-H3	2	0.54
	IFVB	3	0.81
**Paramyxoviruses**		245	66.22
	**RSV**	189	51.08 (45.5-55.9)
	RSVA	17	4.59
	RSVB	172	46.49
	hMPV	32	8.65
	Parainfluenza viruses (PIVs)	67	18.11
	PIV4	2	0.54
	**PIV3**	57	15.41 (11.4-18.4)
	PIV2	3	0.81
	PIV1	6	1.62
**Coronaviruses(CoVs)**		25	6.76
	hCoV- 229E	3	0.81
	hCoV -HKU1	4	1.08
	hCoV- NL63	4	1.08
	hCoV -OC43	16	4.32
**Piconaviruse(PIC)**	**EV/RV**	200	54.05 (48.5-58.9)
**Adenovirus**	**ADV**	48	12.97 (9.5-16.2)
**Bocavirus**	**BoCA**	125	33.78 (28.9-38.4)

The clinical symptoms of all 370 participants presented with different severe respiratory infection signs including convulsion, shock, pleural effusion, cough, wet rale, dry rale, expectoration, vomiting, respiratory failure, and heart failure. The three most commonly observed symptoms were cough (97.57%), wet rale (62.43%), and expectoration (54.32%). 341 participants showed parenchymal infiltration in chest radiography (data not shown).

### Viral Etiology and Seasonal Distribution of Respiratory Viruses

Viral prevalence is also presented in Table 1. Paramyxoviruses have the highest detection rate (66.22%), including RSV51.08%(A, 4.59%; B, 46.49%) (95%CI, 45.5-55.9%); hMPV, 8.65%; and hPIVs, 18.11%((especially PIV3, 15.41%; 95%CI, 11.4-18.4%)), followed by EV/RV (54.05%, 95% CI 48.5-58.9%), BoCV (33.78%, 95% CI 28.9-38.4%), ADV (12.97%, 95% CI 9.5-16.2%), Orthomyxoviruses (7.84%, IFVA,7.03%; IFVB, 0.81%) and hCoVs (6.76%: HCoV-OC43, 4.32%; HCoV-229E, 0.81%; HCoV-NL63, 1.08%; and HCoV-HKU1, 1.08%). Pandemic H1N1 was the dominant influenza virus(IFV) but only detected in 20 (5.41%) of children enrolled in this study. We noticed that all pandemic H1N1 infection took place during 2009(from January through August).

**Figure 1:**
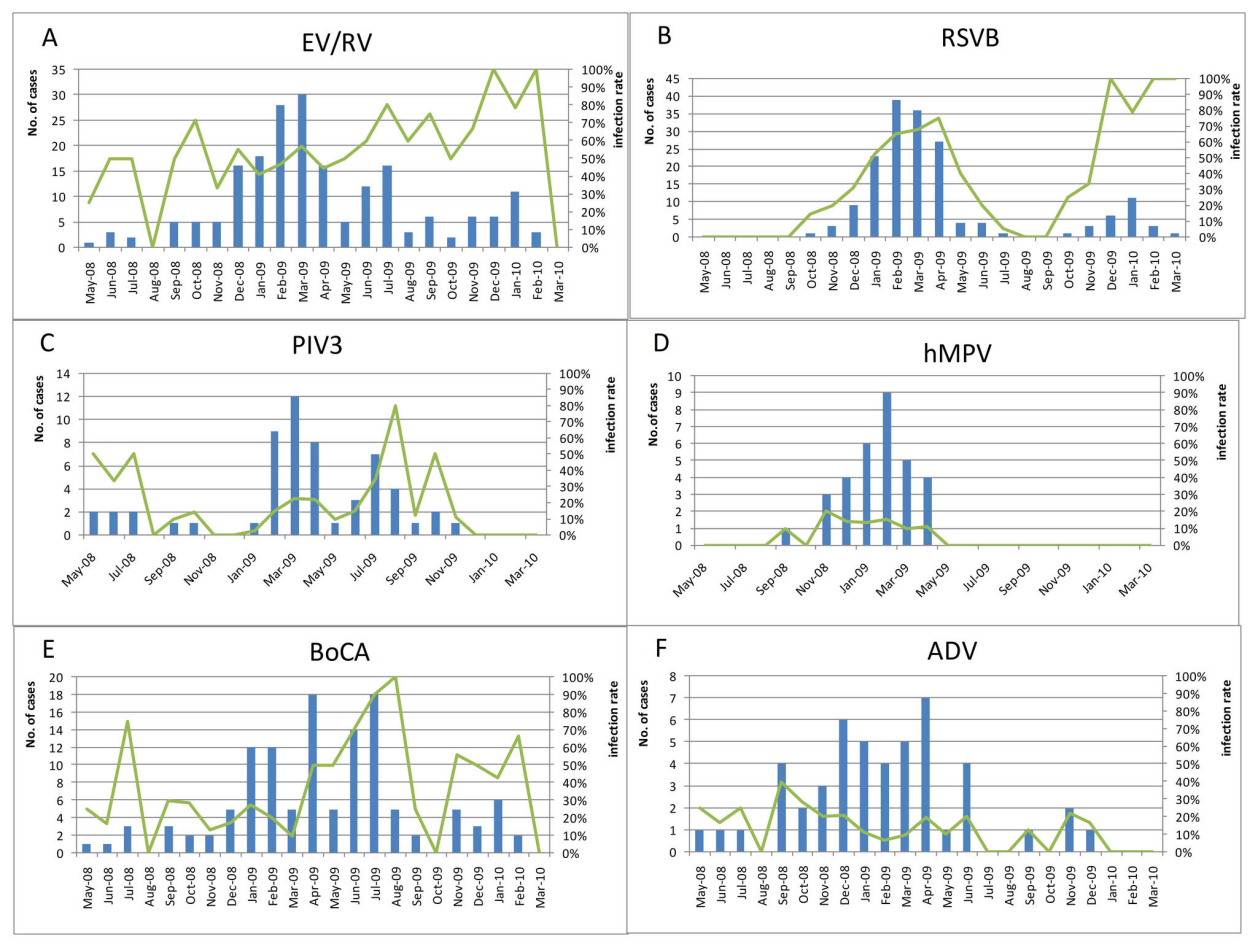
Seasonal distribution of major respiratory viruses: A) EV/RV, B) RSVB, C) PIV3, D) hMPV, E) BoCA F) ADV, in children with ALRI of Beijing Children’s Hospital during September 2008 till March 2010. Primary y-axis and bars describes number of cases, while secondary y-axis and lines describes infection rate.

Figure 1 shows the seasonal distribution of few dominant respiratory viruses and incidence of SARI. EV/RV was the most frequently discovered pathogen in this study; it was active throughout whole year, with a flat line of infection rate around 50% (Figure 1A). RSVB, in contrast to EV/RV, only prevailed in winter. It infected 60-100% patients in winter, but almost disappeared in summer (Figure 1B). hMPV showed similar distribution as RSVB, but the infection rate was much lower (Figure 1D). Of all the other 3 dominant pathogens: both PIV3 (Figure 1C) and BoCA(Figure 1E) showed a peak in the August of 2009, however ADV (Figure 1F) infection was always active with a flat line from September of 2008 to December of 2009.

### Comparison between urban and suburban/rural patients

**Figure 2:**
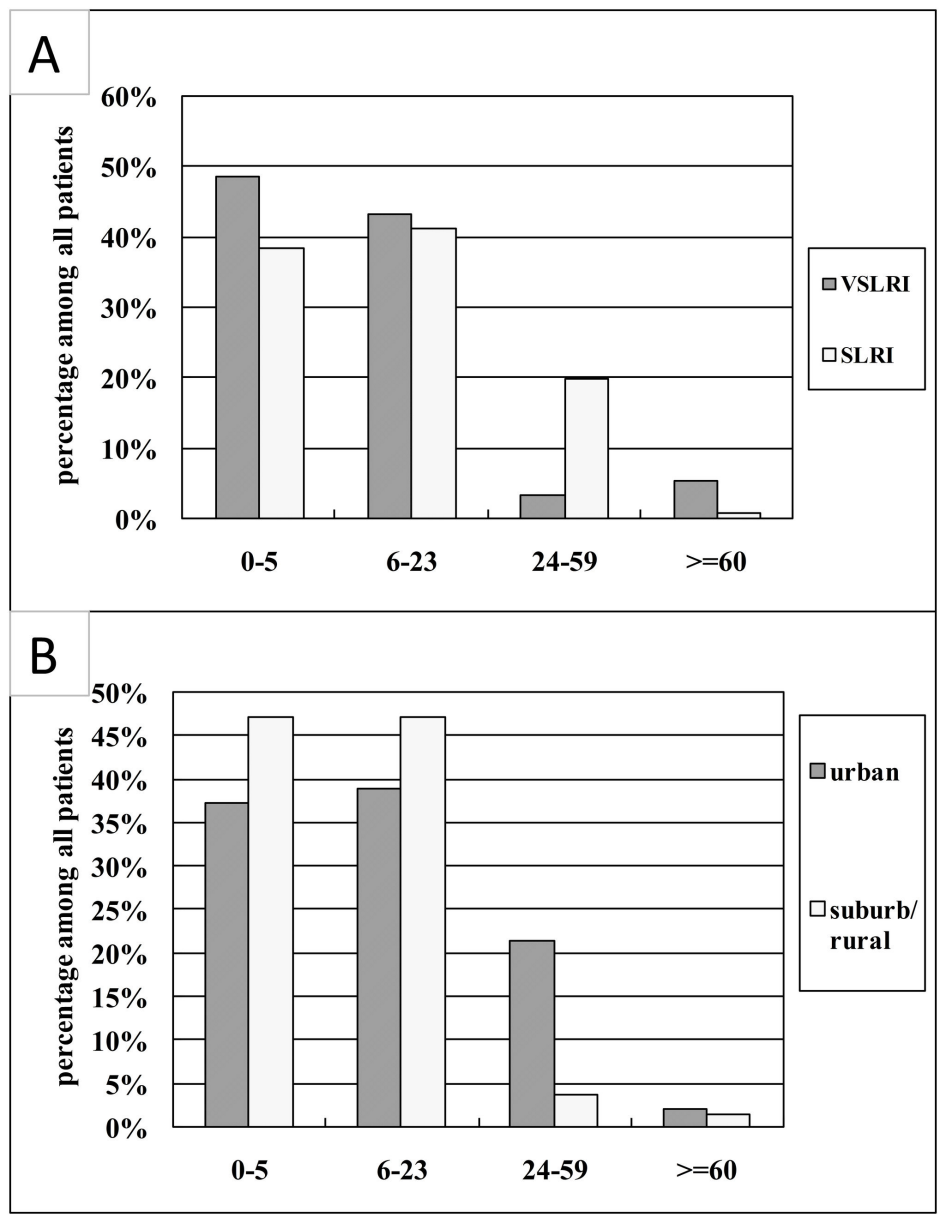
Comparison of age distribution among four age groups based on percentage of patients’ number. A) Comparison between urban and suburb/rural patients. B) Comparison between VSARI and SARI patients.

Patients from urban area are obviously older than suburb/rural ones, average age 15.34 vs. 8.72 months (Table 2). Further analysis of age distribution based on chi-test showed that ratio of young children (24 to 59 months) is obviously higher in rural area (21.6% vs. 3.9%, *P* < 0.001, Figure 2A).

**Table 2:** Comparison between urban and suburb cases.

		**No. (%) of Urban patients**	**No. (%) of Suburb/rural patients**	*P* value*
**Age mean**		15.34	8.72	7.96E-05
**Viral etiology**	RSVA	7 (2.9)	10 (7.8)	0.03
	RSVB	104 (43.2)	68 (52.7)	0.08
	RSV	109 (45.2)	75 (58.1)	0.02
	RSV only	34 (14.1)	21 (16.3)	0.58
	RSV+ other viruses	75 (31.1)	54 (41.9)	0.04
	Non RSV	116 (48.1)	50 (38.8)	0.08
	INFA	19 (7.9)	7 (5.4)	0.39
	H1N1	13 (5.4)	7 (5.4)	0.99
	hCoV-OC43	12 (5)	4 (3.1)	0.40
	PIV3	39 (16.2)	18 (14)	0.57
	EV/RV	140 (58.1)	60 (46.5)	0.03
	hMPV	14 (5.8)	18 (14)	0.01
	ADV	32 (13.3)	16 (12.4)	0.81
	BoCA	82 (34)	43 (33.3)	0.89
**Clinical sign**	Bronchiolitis	8 (3.3)	11 (8.5)	0.03
	Asthmatic Bronchitis	6 (2.5)	10 (7.8)	0.02
**VSARI**		68 (28.2)	52 (40.3)	0.02

* All comparison between urban and suburb/rural patients were performed using Chi-square test

Several viruses are more frequently detected among suburb/rural patients(Table 2), including RSV (urban, 45.2%, suburb/rural, 58.1%, *P*=0.02), especially RSVA (urban, 2.9%, suburb/rural, 7.8%, *P*=0.03), and RSV co-infected with other viruses (urban, 31.1%, 75/241, suburb/rural, 41.9%, 54/129*, P*=0.04), as well as hMPV (urban, 5.8%, 14/241, suburb/rural, 14%, 18/129, *P*=0.01).

Detection of piconaviruses (EV/RV), on the contrary, is higher in urban area (Urban, 58.1%, 140/241, suburb/rural, 46.5%, 60/129, *P*=0.03).

Clinical diagnosis of common virus related respiratory diseases was also compared. Incidence of capillary bronchitis (3.3% vs. 8.5%, *P*=0.03), and asthmatic bronchitis (2.5% vs. 7.8%, *P* =0.02) is higher in suburb/rural area (Table 2). No significant difference was detected among other diseases (data not shown). Occurrence of VSARI is obviously higher in suburb/rural area (40.3% vs. 28.2%, *P*=0.02).

### Comparison between VSARI and SARI patients

To find the reason that causes severe infection, we performed complete comparison between VSARI patients and the SARI, including clinical signs, number of viral target, gender, and age(Table 3, Figure 2B).

**Table 3:** Comparison between VSARI and SARI patients.

		**No. (%) of all samples**	**No. (%) of VSARI**	**No. (%) of SARI**	***Pvalue***
No. of viral target	0	20 (5.41)	7 (7.37)	13 (4.73)	0.06^a^
	1	112 (30.27)	20 (21.05)	92 (33.45)	
	> = 2	238 (64.32)	68 (71.58)	170 (61.82)	
Gender	Female	147 (39.73)	36 (37.89)	111 (40.36)	0.67^*a*^
	Male	223 (60.27)	59 (62.11)	164 (59.64)	
Age mean(month)	average	13.03	10.48	13.9	0.06^*b*^
Total		370	95	275	

*a* Comparison of numbers and proportions were performed using chi-square test

*b* Comparison of continuous variables were performed using variance analysis

No difference in gender or average age was discovered between VSARI and SARI patients. Also multiple infection does not have significant impact in severity of infection (*P*=0.11). However, comparison of age group showed some difference (*P* = 6.93E-05). Judging from detailed list of Figure 2B, it is obvious that patients older than 24 months are more resistant to very severe infection (3.16% vs. 19.64%).

### Association between Viral Etiology and Clinical Characterization among Hospitalized Children with SARI

To find the association between virus infection and clinical signs in SARI, binary logistic regression was performed between 4 commonly diagnosed respiratory abnormality, including anhelation, respiratory failure, heart failure and pleural effusion, and the viral target detected by xTAG® RVP FAST. As shown in Table 4, IFVA is significantly associated with anhelation (*P*=0.03, odds ratio (OR) = 12.5; 95%CI, 12.0-100). PIV3 is also associated with anhelation (*P*=0.02, OR=2.27; 95%CI, 1.16-4.55). HCoV-OC43 is associated with pleural effusion (*P*=0.01, OR=16.67; 95%CI, 1.85-100). hMPV is associated with all three symptoms of severe infection: respiratory failure (*P*=0.01, OR=4.35, 95%CI, 1.39-14.29), anhelation (*P*=0.029, OR=2.49; 95%CI, 1.10-5.65), and heart failure (*P*=0.03, OR=4.35; 95%CI, 1.12-16.67).

Association between commonly diagnosed respiratory disease and virus detection was also performed by binary logistic regression. As shown in Table 5, both RSVA (*P*=0.00, OR=11.11; 95%CI, 2.08-50) and RSVB (*P*=0.00, OR=10; 95%CI, 2.38-50) is associated with bronchiolitis and hMPV associated with Pneumonia/Bronchopneumonia (*P*=0.00, OR=14.29, 95%CI, 2.78-100).

## Discussion

In this study, we performed systematic study of viral etiology and clinical profiles among children with SARI admitted in Beijing Children’s Hospital between May 2008 and March 2010.

Unlike most previous studies in China, which primarily focused on common acute respiratory infections in children [20–23], we exclusively studied the in-hospital patients with deeper infection in the respiratory tract and severer symptoms. 32.34% of the cases in this study suffered very severe symptoms such as heart failure and respiratory failure, as well as complications like diarrhea and vomiting. Another result that is different from previous study is a co-infection rate of 64.32%. Besides the special focus on in-patients, a broader viral spectrum and higher sensitivity of xTAG® RVP FAST test may also give rise to this [15–18,24]. The most supportive evidence is an unprecedented high infection rates of EV/RV (54.05%), which is consistent with previous report that xTAG RVP FAST is extremely sensitive in EV/RV detection compared to other molecular based methods [19,25].

WHO and the Pan American Health Organization recommend hospital-based surveillance of severe acute respiratory infections (SARI) as a tool to monitor severe disease caused by influenza. Although the study was performed during 2009 H1N1 pandemic period, our data showed that the special cohort of SARI patients resulted in relatively low incidence of influenza viruses (7.03%). Pandemic H1N1 was the dominant influenza virus(IFV) but was only detected in 20 (5.41%) of children. As reported in previous study [5,26], children infected with influenza viruses are usually older than those suffered from RSV, while more than 80% patients in this study were under 24 months old.

Paramyxoviruses, especially RSV and hMPV, are might prevalence and might be the main reason of SARI outbreak during 2008 winter to 2009 spring in this study. Similar to previous studies [5,27–29], RSV played an important role in children of SARI. It showed obvious seasonal distribution and was obviously related to several respiratory symptoms. Infection of RSVB increased and peaked with increase of SARI cases during September 2008 through March 2009. Both RSVA and RSVB increased the risk of bronchiolitis with high odds ratio (Table 5). The obvious relationship between RSV and severe respiratory symptoms coincides with a similar retrospective study performed on Kenya children of severe pneumonia, in which RSV, among all commonly discovered respiratory viruses, was found to be the only associated virus with children pneumonia [7,30].

**Table 5:** Association between respiratory viruses and clinical diagnosis.

	**Bronchiolitis**				**Bronchopneumonia/pneumonia**			
	**Sig.** ^a^	**OR** ^b^	**95% CI for OR** ^c^		**Sig.** ^a^	**OR** ^b^	**95% CI for OR** ^c^	
			Lower	Upper			Lower	Upper
**IFVA**	1.00	8.94E-09	.	0.00	1.00	2.05E-09	.	0.00
**RSVA**	**0.00**	**11.11**	**2.08**	**50**	0.87	1.16	0.18	7.14
**RSVB**	**0.00**	**10**	**2.38**	**50**	0.76	1.41	0.16	12.50
**RSV**	**0.00**	**14.29**	**2.78**	**100**	0.83	0.78	0.08	7.69
**hCoV OC43**	0.55	2.13	0.18	25	0.93	1.05	0.30	3.70
**PIV3**	0.78	0.79	0.15	4.17	0.72	1.12	0.60	2.08
**RV/EV**	0.80	0.87	0.30	2.56	0.12	0.69	0.44	1.10
**hMPV**	0.26	2.63	0.48	14.29	**0.05**	**2.17**	**1.00**	**4.55**
**ADV**	0.91	1.10	0.21	5.88	0.19	0.63	0.31	1.27
**BoCA**	0.63	0.74	0.21	2.63	0.03	0.57	0.34	0.94

*a*: *P* value of binary logistic regression analysis between respiratory virus and clinical diagnosis

*b*: odds ratio of binary logistic regression analysis between respiratory virus and clinical diagnosis

*c*: 95% confidence interval of binary logistic regression analysis between respiratory virus and diagnosis

Another paramyxoviruse, hMPV, showed similar seasonality as RSVB, although the infection rate was not high among all cases. What’s more important is that infection of hMPV is obviously related to all three VSRI-related symptoms: anhelation, heart failure, and respiratory failure (Table 4).

**Table 4:** Association between respiratory viruses and clinical signs.

	**Anhelation**				**Respiratory failure**				**Heart failure**				**Pleural effusion**				
	**Sig.** ^a^	**OR** ^b^	**95% CI for OR** ^*c*^		**Sig.** ^a^	**OR** ^b^	**95% CI for OR** ^*c*^		**Sig.** ^a^	**OR** ^b^	**95% CI for OR** ^*c*^			**Sig.** ^a^	**OR** ^b^	**95% CI for OR** ^*c*^	
			Lower	Upper			Lower	Upper			Lower	Upper				Lower	Upper
**IFVA**	**0.03**	**12.5**	**12.20**	**100**	0.13	7.69	0.55	100	1.00	3.83E-08	.	0.00		0.25	7.69	0.25	0.00
RSVA	0.84	0.79	0.08	7.69	1.00	6.57E-09	.	0.00	1.00	1.12E-08	.	0.00		1.00	1.72E-07	.	0.00
RSVB	0.46	0.37	0.03	5.00	1.00	1.46E-09	.	0.00	1.00	1.02	.	0.00		1.00	2.27	.	0.00
RSV	0.29	0.24	0.02	3.45	1.00	7.87E-10	.	0.00	1.00	0.65	.	0.00		1.00	0.14	.	0.00
**hCoV OC43**	0.79	1.19	0.33	4.17	0.49	0.43	0.04	5.00	1.00	0.14E-08	.	0.00		**0.01**	**16.67**	**1.85**	**100**
**PIV3**	**0.02**	**2.27**	**1.16**	**4.55**	0.12	2.17	0.81	5.88	0.55	1.45	0.43	5.00		0.61	0.51	0.04	6.67
RV/EV	0.73	0.89	0.47	1.69	0.92	1.05	0.40	2.78	0.78	0.85	0.29	2.70		0.08	0.16	0.02	1.27
**hMPV**	**.029**	**2.49**	**1.10**	**5.65**	**0.01**	**4.35**	**1.39**	**14.29**	**0.03**	**4.35**	**1.12**	**16.67**		1.00	5.00E-08	.	0.00
ADV	0.35	0.66	0.28	1.56	0.91	1.08	0.32	3.70	0.65	1.39	0.34	5.56		0.16	3.85	0.60	25
BoCA	0.63	1.15	2.00	0.65	0.83	1.10	2.56	0.47	0.50	0.70	4.00	0.51		0.13	5.88	50.00	0.57

*a*: *P* value of binary logistic regression analysis between respiratory virus and clinical signs

*b*: odds ratio of binary logistic regression analysis between respiratory virus and clinical signs

*c*: 95% confidence interval of binary logistic regression analysis between respiratory virus and clinical signs

The third commonly detected paramyxovirus was PIV3, a viral target infects about 1-13% of children with pulmonary diseases. Infection rate varies with age and severity of cohort among different studies [5,31]. PIV3 is the predominant subtype among parainfluenza viruses, and was responsible for 83.8% parainfluenza infection in this cohort. To our knowledge, PIV3 was more related with immunocompromised children than common SARI patients in previous studies [32–35].

It has frequently been reported that infection of RSV and hMPV result in same or similar symptoms and were indistinguishable on clinical basis [9–11,36,37]. However, our analysis of association produced somehow different results: hMPV was more directly related with VSARI and thus improved risk of pneumonia, while RSV was more related to risk of bronchiolitis.

In contrast to paramyxoviruses, infection rate of EV/RV distributed relatively even throughout the whole year, in spite of its high total detection rate (50.4%). This assumption is supported by association study, in which no confident relationship was found between EV/RV and any severe symptoms (Table 4) or any diagnosis (Table 5). In reference to previous studies, EV/RV in respiratory tract is frequently detected by PCR among children of upper respiratory infection and asymptomatic controls. However, few evidence has been found supporting association between EV/RV detection and severe lower respiratory symptoms [36,37]. Further studies are needed to address the relevance of the EV/RV single and coinfections on clinical outcomes.

Great difference was discovered between urban and suburb patients in this study. Children from suburb area are apparently younger, with higher incidence of SARI, and were more susceptible to RSV, hMPV, and EV/RV. Bronchiolitis and asthmatic Bronchitis also happened more frequently in suburb area (Table 2).

For each of the five most popular pathogens: BoCA, ADV, RSV, EV/RV (piconaviruses), and PIV3, we performed binary logistic regression between existence of co-infection and all clinical signs. Unfortunately, no statistical significance was observed in any run of regression（data not shown).

To our knowledge, this is the first report on the viral etiology, epidemiological and clinical profiles of hospitalized children with SARI using a commercial assay for 18 respiratory viral targets in Asia. Improved laboratory test highlighted the significance of viral etiology and its distribution among children with SARI in China. Our data is similar to the recent surveillance report on SARI in South Africa by use of the validated RT-PCR multiplex assay(39), which is also consistent with several studies in other parts of the world(3,7,30). Secondary, Comparison analysis was firstly performed between urban and suburban/rural patients in china; In addition, this is also the first study on association analysis between viral etiology and clinical profile among VSARI and SARI patients. Our data first indicated that hMPV was more directly related with SARI and thus improved risk of pneumonia. Association viral etiology with clinical outcome might assist clinicians in appropriately treating hospitalized children with SARI.
